# “Land-sparing benefits biodiversity while land-sharing benefits ecosystem services”: Stakeholders’ perspectives on biodiversity conservation strategies in boreal forests

**DOI:** 10.1007/s13280-023-01926-0

**Published:** 2023-10-11

**Authors:** Therese Löfroth, Sonia Merinero, Johanna Johansson, Eva-Maria Nordström, Emma Sahlström, Jörgen Sjögren, Thomas Ranius

**Affiliations:** 1 Department of Wildlife, Fish and Environmental Studies, Swedish University of Agricultural Sciences, 901 83 Umeå, Sweden; 2 Departamento de Biología y Geología, Física y Química Inorgánica, Universidad Rey Juan Carlos, C/Tulipán s/n, 28933 Móstoles, Spain; 3 School of Natural Sciences, Technology and Environmental Studies, Södertörn University, 141 89 Huddinge, Sweden; 4 Department of Forest Resource Management, Swedish University of Agricultural Sciences, 901 83 Umeå, Sweden; 5 Department of Urban and Rural Development, Swedish University of Agricultural Sciences, Box 7012, 750 07 Uppsala, Sweden; 6 Department of Ecology, Swedish University of Agricultural Sciences, Box 7044, 750 07 Uppsala, Sweden

**Keywords:** Forestry, Nature reserves, Scenario analysis, SLOSS, Stakeholder analysis

## Abstract

Biodiversity conservation and economic profit from forests can be combined by various land-sparing and land-sharing approaches. Using a semi-structured survey, we evaluated support for scenarios representing contrasting conservation strategies in a managed boreal forest landscape. Land-sparing approaches were supported by the conservation organisation, regional administrations and the forest company, mainly motivated by the benefit for biodiversity based on ecological theory. Land-sharing approaches were supported by one recreational organisation, some municipalities and the forest owners’ association, mainly motivated by the delivery of ecosystem services. Stakeholder groups using certain ecosystem services had motivations that we related to an anthropocentric mindset, while others focused more on species conservation, which can be related both to an anthropocentric or an ecocentric mindsets. Forest conservation planning should consider stakeholders’ preferences to handle land-use conflicts. Since reaching consensus among multiple stakeholders seems unfeasible, a combination of land-sparing and land-sharing approaches is probably the best compromise.

**Supplementary Information:**

The online version contains supplementary material available at 10.1007/s13280-023-01926-0.

## Introduction

More than 2 billion hectares of the world's forests (approximately 55% of all forest area) are managed as production forests or used to extract multiple values (FAO 2010). Production forestry shapes forest landscapes by increasing the proportion of young forest, altering tree species composition and increasing stand homogeneity (Paillet et al. 2010; Horstkotte et al. 2016; Naumov et al. 2018; Rodriguez et al. 2021). These changes has negative effects on biodiversity (Venier et al. 2014) and also on the provision of many ecosystem services, such as opportunities for recreation (Edwards et al. 2012). Where several stakeholders use the same land for different purposes, trade-offs in management goals are unavoidable, e.g., commercial extraction of resources versus social, cultural and biological values (Wiens 2008; Horstkotte et al. 2016). This is particularly relevant in countries where forests are managed on a significant share of the landscape.

Biodiversity can be preserved by setting aside large unmanaged areas as well as by maintaining habitats and structures for biodiversity in managed forests (Mell 2017; Law et al. 2021). Usually, these two approaches are combined, and this is done in different ways in different parts of the world (Betts et al. 2021). Sometimes managed forests are divided into intensively and extensively managed stands (Cote et al. 2010). The amount of protected forest as well as the levels of retention of key habitat features (e.g., dead and living trees left after felling, buffer zones around watersheds) in managed forest varies among countries. For example, the area or wood volume retained after harvesting vary considerably, from 1–3 percent of the harvested volume in Finland to more than 40 percent in parts of Vancouver Island (Gustafsson et al. 2012).

In Sweden, multi-scale conservation is applied (Gustafsson and Perhans 2010). This approach combines large protected areas (e.g., national parks), intermediate-scale reserves set within the production forest matrix, and at the smallest scale, retention of key habitat features within production stands (Lindenmayer et al. 2006; Gustafsson and Perhans 2010). This approach is applied in forest landscapes characterized by a long management history with remaining natural habitat highly fragmented. In more intact forest landscapes, protection of larger forest areas should be a more dominating part of the conservation strategy to reach biodiversity conservation goals (Ranius and Kindvall 2006).

Swedish forest policy is multi-objective by promoting economic benefits, biodiversity conservation and recreation values, involving a wide range of stakeholders with different values and conflicting perspectives on forest management (Sandström et al. 2016; Beland Lindahl et al. 2017). Theoretically, rational and well-informed actions by individual forest owners, consumers and other market actors are expected to result in actions along a desired way of development (Roberge et al. 2020). However, research shows that there are weaknesses with this policy: forest owners are assumed to have “freedom with responsibility” but actors diverge about the meaning of this term (Löfmarck et al. 2017), biodiversity goals are not met (Angelstam et al. 2020) and the biodiversity protection measures generate habitat amounts that are far below assumed thresholds and amounts in natural habitats (Angelstam et al. 2013; Johansson et al. 2013). While the current management practices (rotation forestry) favour wood production, biodiversity-oriented management (extended rotation periods, more set-asides, and continuous cover forestry, and ecological landscape planning) are needed to fulfil ecological and social goals (Michanek et al. 2018; Eggers et al. 2019; Felton et al. 2020; Bostedt et al. 2021). As a result of the different interests, perspectives, rights and powers among stakeholders, stakeholders strongly differ in their opinions regarding current and future forest management practices, including how to balance the forests’ economic, environmental, social and esthetic values (Sandström et al. 2016).

The aim of this study was to understand the preferences and underlying values of different stakeholders for conservation strategies ranging from land-sparing to land-sharing approaches in boreal forests. Specifically, we used a semi-structured survey to compare support for i) biodiversity-oriented management in managed stands or area protection, ii) spatial allocation of conservation measures, and iii) protected areas of different sizes. To understand their underlying values, stakeholders provided a written statement about their main source for knowledge acquisition. Finally, we discussed how our results might contribute to a way forward in the heated debate on forest conservation and management.

## Concepts and theory

### Land-sparing versus land-sharing

Biodiversity conservation measures can be classified in two main strategies depending on their spatial allocation and type of management (Phalan et al. 2011). First, land-sparing implies spatial separation of conservation areas from agricultural or forest production areas. Second, land-sharing implies that multiple goals (e.g., production and conservation) are integrated within sites (Phalan et al. 2011). At a landscape scale, land-sparing consists of a distinct zonation where some parts of the landscape are dedicated to either intensive management or protection for conservation purposes, while land-sharing consists of conservation measures more evenly or randomly distributed in the landscape. Which of these strategies that is most beneficial for biodiversity is still under debate (Kremen 2015; Grass et al. 2021). According to ecological theories, a land-sparing strategy could favor biodiversity because larger habitat patches host larger populations, decreasing the risk of local extinctions (Hanski 2000). Hanski (2011) introduced the concept of “Third-of-a-third” implying that in a third of the managed landscape and in addition to areas protected in formal reserves and national parks, a third of the land should be set aside for conservation, arguing that set aside areas concentrated in part of the landscape allow dispersal of individuals among set asides and viable populations. Land-sharing may be favourable if the goal is both production and maintenance of biodiversity across the whole landscape (Mell 2017). The outcome also depends on the intensity of forest management in the production stands. The land-sparing/land-sharing framework was originally developed for agricultural land and has been rarely applied in forest land (see however, Paul and Knoke 2015; Betts et al. 2021). In forests, land-sharing have the potential to be more beneficial than in agricultural landscapes because managed forests can be more similar to natural habitats than crop fields. However, the outcomes of the strategy in forest land vary depending on e.g., management history, climate, and social context (Sterling et al. 2017; Naumov et al. 2018).

Here, we define land-sparing as conservation strategies where wood production is separated from biodiversity conservation by area protection and where area protection in the landscape is concentrated. Land-sharing is defined as the combination of wood production and biodiversity conservation in the same stands either by leaving retention patches or by extended rotations.

### Forest policy

Globally, there is an increased demand for wood products and thus, there is an increased pressure to produce more of them (Kok et al. 2018). At the same time, there are clear political goals to halt deforestation and to conduct forestry in sustainable ways. This is put forward globally in the UN strategic plan for forests (DESA 2019) and at European level in the EU forest strategies (Larsen et al. 2022). Regarding biomass extraction, forestry in Fennoscandia is one of the most efficient in the world. In Sweden, forestry is applied on 94% of the productive forest land (i.e., land that has the capacity to produce > 1 m^3^ ha^−1^ year^−1^). Rotation forestry, including harvesting of mature stands followed by soil scarification, planting and thinnings before the stand is harvested again, is the dominant management method. Other management regimes such as continuous cover forestry or nature-based forest management is only practiced on a small proportion of the land. This is in sharp contrast to other regions, such as central Europe, where nature-based forest management is the dominating management method (Mason et al. 2021; Larsen et al. 2022).

Approximately 9% of the Swedish forest land is formally protected in national parks, reserves and smaller set-asides (SEPA 2023). On productive forest land certified under FSC or PEFC another 5% is protected in voluntary set asides (FSC, 2020; PEFC, 2017). In these protected areas, typically no commercial wood extraction is allowed. These areas are unevenly distributed in Sweden; most protected areas are situated in low-fertile areas close to the northwest mountain range (Svensson et al. 2020). In more productive areas further east and south much smaller proportions are protected. In addition, forest owners certified under Forest Stewardship Council (FSC) or Program for Endorsement of Forest Certification (PEFC) are obliged to set aside 5% of their forest land for biodiversity (voluntary set asides). In managed stands certified by Forest Stewardship Council (FSC), an average of 9% of the standing volume is retained as key habitat features at clear felling (Eriksson et al. 2007). This retention is partly a consequence of legislation, and partly a voluntary measure. Forest management is regulated by the Forestry Act, which since 1993 establishes two equally important goals: (i) sustainable production of timber (and other products) to ensure economic growth; and (ii) biodiversity conservation. However, the Act only prescribes minimum criteria related to these goals and does not stipulate detailed practices for meeting them. Instead, Swedish forest policy affirms the importance of “freedom with responsibility”, granting forest owners substantial scope to decide how to incorporate biodiversity protection measures in their forests (Beland Lindahl et al. 2017; Löfmarck et al. 2017). Several voluntary measures initiated by landowners are subsidised by authorities, such as protection of small biotopes, rewetting of drained areas, and promotion of deciduous trees. Recently, the policy of area protection has been changed, so it should primarily be initiated by land owners and not by authorities (Anonymous 2021).

### Understanding underlying values

Values denote how people articulate, systematize and defend preferences that guide their behavior. Values thus influence how humans think about and interact with their surroundings (Manfredo et al. 2009). Therefore, it is important to understand not only the arguments for different conservation strategies but also the underlying values forming those arguments. Perspectives around the treatment of nature can be divided into anthropocentrism and ecocentrism (Taylor 2005). In an anthropocentric ethic, nature deserves moral consideration because it has instrumental value to humans, through ecosystem services, while an ecocentric ethic postulates that nature deserves moral consideration because it has an intrinsic value (Kortenkamp and Moore 2001). Therefore, ecocentrism is often associated with support for conservation actions even at the expense of human interests (Dietsch et al. 2016). In the Swedish Forestry Act, the environmental concern is mainly focused on biodiversity conservation. There are many reasons for preserving biodiversity, including both its instrumental values, and ecocentric arguments (Brown 2001). Later, the environmental concern in Sweden has largely extended to provide ecosystem services (Hysing 2021). The ecosystem service concept is anthropocentric, considering only the instrumental value of nature for humans (Silvertown 2015). Thus, within Swedish forest policy, there is a gradient from purely anthropocentric perspectives (represented by the ecosystem service concept) to perspectives that can be supported also from an ecocentric viewpoint (represented by biodiversity conservation).

### Methods and case study

First, we developed seven biodiversity conservation scenarios set on a managed Swedish forest landscape. Second, we used a semi-structured survey where stakeholders evaluated the resulting spatial configuration of conservation areas and forest characteristics (i.e., stand age, volume of large trees, and amount of deadwood) from each scenario across the landscape. Finally, we analysed their answers in a land-sparing/land-sharing framework to evaluate which scenarios were preferred by different stakeholders and the motivations for their preferences.

### Scenario simulations

We projected seven conservation scenarios in a typical managed boreal forest landscape in Sweden over a 100 year period using the Heureka system (Wikström et al. 2011). The studied landscape comprises around 15 000 ha of managed productive forest (i.e., with a potential mean annual increment > 1 m^3^ha^−1^) and is located in central Sweden (Delsbo, 62° N, 16° E, altitude: 140–530 m). Scots pine (*Pinus sylvestris* L.) and Norway spruce (*Picea abies* (L.) Karst.) make up 50% and 33% of the total standing volume, respectively. Since the 1950s, the forest has been managed by rotation forestry which has promoted even-aged, conifer dominated stands. Today the average stand age is 45 years. About 80% of the productive forest is younger than 60 years, and 10% of the productive forest is older than 100 years. The forest owner has been certified according to the Forest Stewardship Council since the late 1990s, and thus follows the requirements to retain trees during at clear-felling and leave ≥ 5% of the productive forest land unmanaged in set asides (FSC 2010).

All scenarios applied the same level of conservation effort (same proportion of unmanaged area within a managed forest landscape, or, for extended rotation, the same long-term economic outcome). Four scenarios represented land-sparing options resulting from the combination of different size (large and small) and spatial allocation (dispersed and concentrated) of protected areas and three scenarios represented land-sharing options either through small groups of retention trees (either dispersed or concentrated groups of retention trees) or extended rotation length. In each scenario, 16% of the area was dedicated to biodiversity conservation. This level represents the proportion of land not used for outtake of wood within productive forest (including formally protected land, voluntarily set-aside stands, and retention) presently both in Sweden and in Gävleborg, the county where the landscape is located (Claesson et al. 2015, for Gävleborg: data from “SKA analyses” performed by the Swedish Forest Agency).

All seven scenarios shared a baseline of 6% of unmanaged area, comprising areas of different sizes (large and small protected areas and groups of retention trees) equally distributed around the landscape. The scenarios differed in the dominant conservation measure and its allocation in the landscape providing the additional 10% of the total area (see Fig. S1). In six scenarios, the dominant conservation measure was one of the three sizes of unmanaged areas, either dispersed around the whole landscape or concentrated in a part of the landscape. In the seventh scenario, in addition to the baseline, rotation length of managed stands was extended so that the net present value was similar to that of the other six scenarios. Consequently, the minimum final felling age in this scenario was set to be at least 1.5 times the lowest allowable final felling age. The lowest allowable felling age depends on site productivity, and varied between 45 and 90 years in the studied landscape (for details see Filyushkina et al. (2022)).

Scenarios were built, simulated, and spatially represented in Heureka, a Swedish software for forest management decision support (Wikström et al. 2011). They were created by allocating each forest stand to one of three treatments: no management (for protected areas), conventional even-aged management with groups of retention trees, and extended rotation even-aged management. The choice of the treatment for each stand was based on their current age (at year 0), location in the landscape and area constraints set for each scenario. The oldest stands were prioritised for being selected as “no management” treatments. In the survey, outputs from Heureka in the form of maps depicting mean stand age, volume of deadwood and volume of large trees (diameter > 30 cm) per ha both at year 0 and 100 (at the end of simulation period) were presented to stakeholders (Fig. S1).

### Data collection

We selected stakeholders that are important in Swedish forests and affected by biodiversity conservation measures (Sandström et al. 2016; Eggers et al. 2019). The selected organisations represented different interests, especially forest production, recreation, and biodiversity conservation. Forest companies and the members of forest owners’ associations own and manage forest land and are the foundation of Swedish forestry. They are directly affected by biodiversity conservation measures and also have the power to decide about them on their properties. The regional administrations (county administration boards) are responsible for the establishment and management of protected areas and to provide infrastructure for recreation in protected areas. The recreational organisations were selected among those that use forest for sports and recreational purposes including hiking and hunting. The environmental organisations influence public opinion and decision making for biodiversity conservation. They also act as watchdogs, overseeing and report on actions that negatively impact biodiversity, both generally and on specific sites, for example by reporting logging of sites with high biodiversity values.

Before submitting the survey, we provided a short description of the project aims and asked each organisation to decide their representative answering the survey. Stakeholders evaluated the biodiversity conservation strategies based on the resulting landscapes from each scenario (Supplementary material, S1). To keep the focus on general rather than on local effects of the scenarios, we did not provide the stakeholders with the exact location of the scenario landscape. In April-June 2021, we e-mailed the online semi-structured survey to 45 organisations (5 forest companies, 2 forest owners’ associations, 2 governmental agencies, 13 regional administrations and 13 municipalities, 3 environmental organisations and 7 recreational organisations). In August 2021, we re-submitted the survey to those that did not respond to increase the response rate. In total, we gathered 21 responses (one forest company, one forest owners’ assocations, one environmental organisation, 12 regional administrations and 5 municipalities and 1 recreational organisation). The proportion of respondents in relation to the number of organisations that received the survey was similar for all types of organisations. Since we received at least one answer for each group, we consider them as good examples of the forest stakeholders’ perspectives. However, for those four stakeholder groups with only one respondent we did not get any measure of the variability within a group.

The stakeholders answered all questions via the digital survey-tool Netigate (Netigate AB, Sweden) (Supplementary material, S2). The survey included closed questions about the goal of each organisation, including their priorities within 4 categories (biodiversity conservation, wood production, recreation and climate change mitigation). We asked them to rank the suitability of each scenario on a scale from 1 to 5 according to their organisation’s goals, and to select their most and least preferred scenario. We used an ordinal answer scale which is a widely used rating system in social sciences questionnaires (Joshi et al. 2015). Our scale captured a gradient between two negative and two positive responses, providing a neutral response in between. After all closed questions, respondents could add free text comments about the motivations for their choices of the least and most preferred scenario underlying such preferences. Out of the 21 responses, we compiled all arguments and selected citations for the preferred scenarios that were related to conservation in managed stands versus area protection, concentrated versus dispersed allocation, and size of protected areas.

## Results

The responses on the survey show that the forest company, the regional administrations and the environmental organisation rated alternatives including area protection as more suitable options than tree retention within managed stands or extended rotation. The forest owners’ association and the recreational organisation rated retention and extended rotations as more suitable (Table 1). Stakeholders also had strongly contrasting opinions about the suitability of each scenario and about what was the ‘best’ and ‘worst’ option.

**Table 1:** The goals of the responding organisations (3 = high priority goal, 2 = low priority goal, 1 = not a goal) and the level of support for scenarios for different stakeholder groups (1 is no support, 3 is moderate support and 5 is high support). For groups with several respondents, the numbers are average + - standard deviation, and the range is shown in brackets. The number of respondents is shown in brackets after each respondent

	Forest company	Forest owners’ association	Regional administrations (12)	Municipalities (5)	Recreational organisation	Environmental organisation
**Organisation goals**						
Biodiversity conservation	3	2	3.0 ± 0.0	3.0 ± 0.0	3	3
Wood production	3	2	1.3 ± 0.6	2.0 ± 0.9	1	1
Recreation	3	2	3.0 ± 0.0	3.0 ± 0.0	3	3
Climate change mitigation	3	2	2.7 ± 0.6	2.8 ± 0.4	3	3
**Suitability of scenarios**						
Large dispersed	4	2	3.8 ± 0.7 (3–5)	3.6 ± 1.0 (2–5)	2	4
Large concentrated	5	2	4.4 ± 0.8 (3–5)	3.8 ± 1.2 (2–5)	2	5
Small dispersed	3	3	2.7 ± 0.6 (2–4)	3.6 ± 0.8 (3–5)	3	3
Small concentrated	4	2	3.4 ± 0.8 (2–5)	4.0 ± 0.6 (3–5)	2	3
Retention dispersed	2	4	2.6 ± 0.9 (1–4)	4.0 ± 0.9 (3–5)	4	2
Retention concentrated	3	4	3.4 ± 0.9 (2–5)	3.4 ± 1.2 (2–5)	2	2
Extended rotation	1	3	3.5 ± 1.2 (2–5)	3.6 ± 1.2 (2–5)	5	3
Best scenario	Large concentrated	Retention	Large concentrated	Large concentrated	Extended rotation	Large concentrated
Worst scenario	Extended rotation	Large concentrated	Retention dispersed	Large dispersed	Large	Retention dispersed

### Conservation measures in managed stands versus area protection

Protection of smaller or larger areas (Fig. S1 1A–B, 2A–B) were considered as acceptable or good alternatives (i.e., rated three or higher) by the forest company, regional administrations and the environmental organisation. Conservation measures in managed stands (tree retention groups and extended rotations, Fig. S1 #A-B, 4) were considered acceptable or good alternatives by the forest owners’ association and the recreational organisation (Table 1). Furthermore, preferences for the most and least suitable scenarios varied, and in some cases, the preferred scenario of one stakeholder was the least preferred by another (Table 1). Protecting forest areas was considered the best scenario by the environmental organisation, all regional administrations, three out of five municipalities, and the forest company but was the least preferred by the forest owners’ association, the recreational organisation, and two municipalities. The forest owners’ association and one municipality preferred retention and the recreational organisation and one municipality preferred extended rotations (Fig. S1 4).

Regional administrations and the environmental organisation argued that land-sparing in the form of area protection is better than land-sharing in the form of retention in managed stands or extended rotations. One regional administration argued that formally protected areas are better because they have a greater probability to conserve species over time. Another regional administration acknowledged that protected areas are valuable for both biodiversity and recreation: “*For biodiversity the most important factor is that continuous areas of old forest is protected, but for recreation the availability of forest for recreation is important. Age and natural structures are of great importance for both biodiversity and recreation*”. The forest company argued that land-sparing by area protection helps to focus production forestry to certain areas.

Municipalities used not only biodiversity conservation arguments for area protection (land-sparing) similar to those expressed by the regional administrations, but also recreational arguments: area protection allows them to prioritize recreational areas close to the main city or that a relatively large protected area is used by the public for recreation. For instance, one respondent from a municipality wrote:”*We have a 32 ha reserve that is visited by many inhabitants of the municipality*”.

**Fig. 1:**
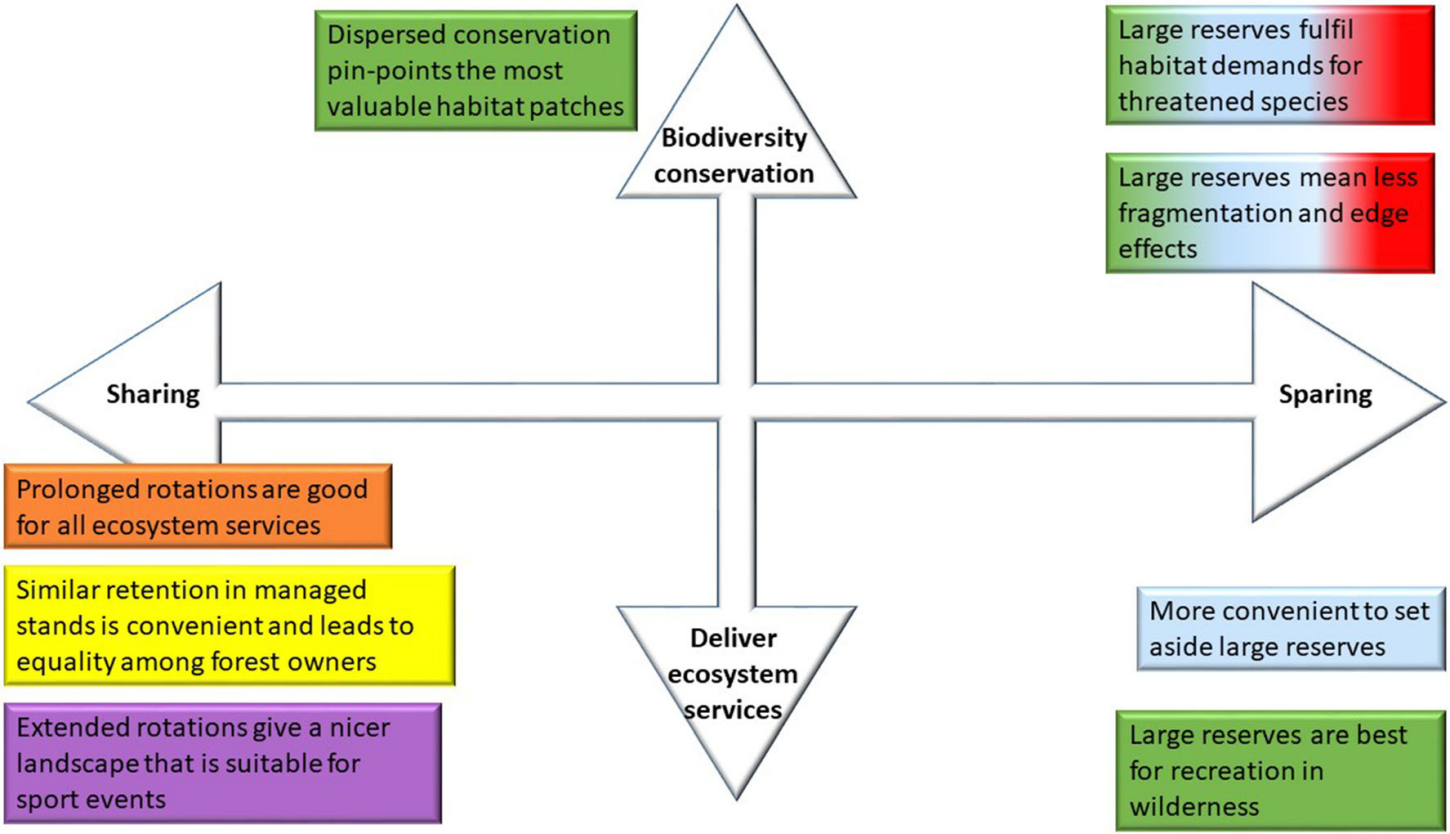
Arguments for land-sharing (i.e., the scenarios combining forestry and conservation in the same area by small groups of retention trees or by extended rotations) and land-sparing (i.e., the scenarios separating management and conservation purposes by area protection) used by stakeholders in our survey. Their placement on the biodiversity conservation/ecosystem service axis is based on our interpretation. The content of the boxes are arguments put forward by the stakeholders and not scientifically evaluated facts. The colors of the boxes represent the stakeholder expressing the arguments. Green = regional administration, orange = municipality, red = environmental organisation, blue = forest company, yellow = forest owners’ association, purple = recreational organisation

Arguments for conservation measures in managed stands (land-sharing) were mostly based on practical, recreational, and esthetical motivations, or arguments related to justice. Justice arguments were put forward from the forest owners’ association: groups of retention trees spread out across the landscape would be “*least negatively affecting forest owners’ freedom and is probably beneficial if the goal is to supply ecosystem services (i.e., good for humans)”*. Retention was also motivated by the straightforward management approach of retaining trees at every cutting: “*Easy and rational to leave small retention groups at each site*”. The recreational organisation motivated land-sharing in the form of extended rotations with esthetical arguments combined with the view that reserves are associated with unwanted regulations: “*It will result in a more interesting landscape for recreation. Reserves can be restrictive because they result in inaccessible biotopes where you need tracks to get around. Many reserves also have restrictions against sport events*”*.* One municipality argued that extended rotation would benefit multiple goals as a result of increased forest age (Fig. 1).

### Spatial allocation of conservation measures

Among the most preferred scenarios, concentrated conservation measures occurred more than twice as often as dispersed allocation. The opposite pattern was shown for the worst scenario. Eight out of 12 regional administrations, three out of five municipalities, and the forest company preferred concentrated allocation. Dispersed allocation was preferred by the forest owners’ association and two municipalities.

Concentrated allocation of protected areas was motivated by species’ dispersal limitations which makes it difficult to cope with habitat loss and fragmentation, exemplified by one regional administration: “*There need to be aggregations of those natural habitats that formerly covered whole areas to conserve organisms associated with these habitat. Enough habitat amount within short enough distances*” and”*[…] by concentrating these [reserves] within a part of the total landscape (approximately 5000 ha) the distance among set aside areas gets smaller, which benefits species limited by dispersal and these species can sustain in viable populations*”. Regional administrations and the forest company referred to specific publications, e.g., about Hanski’s third-of-a-third (Hanski 2011) as expressed by the forest company: “*[…] To concentrate protected areas increases the possibilities to reach critical thresholds for the most endangered species in these landscapes. I think we need to differentiate more based on the thoughts in Hanski’s model third of a third, large protected areas also have less negative impact from edge effects than smaller areas*”.

A municipality also argued that concentrated protected areas might be better for recreation if they are located close to a densely populated area: “*The municipalities’ work with protected areas is mainly focused on securing recreational areas close to the main town*”. Similar arguments were used by one regional administration stating that reserves concentrated to main towns and areas with summer houses give many people access to recreational areas.

Regional administrations argued that dispersed allocation has greater probability to include a wider range of habitats and habitats of higher quality. This was, for example, expressed by one regional administration: “*The reason why I chose them [the reserves] spread in the landscape is that I think that makes it possible for several different natural habitats to be protected than if all set asides are concentrated to a part of the landscape. It is probably easier to find areas of high quality if they are more spread out*”. The forest owners’ association argued that dispersed allocation was more beneficial for ecosystem services “*For biodiversity to be useful for people (ecosystem services) it needs to be present where people are active, i.e., spread out in the managed landscape*”. Several regional administrations and the recreational organisation argued that protected areas spread out in the landscape benefit recreation (Fig. 1).

### Size of protected areas

All regional administrations, three out of five municipalities, the forest company, and the environmental organisation preferred large protected areas. They motivated the choice by ecological theories and claimed that larger reserves imply reduced edge effects and larger population sizes which implies higher long-term persistence of species: “*Larger areas that are not affected means that species that demand large areas or species with low dispersal ability have a chance to persist. Less edge effects* = *higher quality in the protected areas*”. Furthermore, one municipality claimed that “*Large protected areas are always better for biodiversity, recreation and climate—many random factors are evened out*”. A similar way of reasoning was shown by one regional administration: *“Larger areas are more stable over time and more resilient to edge effects, larger functional area*”. The forest company and a municipality argued that recreational activities are easier to perform in large than in small areas. Large reserves were also considered to be more attractive for recreational activities like hiking since they have larger areas of natural habitats. However, for activities such as sporting events, formally protected areas were considered less beneficial due to restrictions of certain activities (Fig. 1).

Motivations for small reserves was that they allow many people to access recreation areas. However, small reserves and groups of retention trees were more often mentioned as the worst scenario as these were considered less beneficial for biodiversity.

## Discussion

### Conservation measures in managed stands versus area protection

Stakeholders that argued for area protection (land-sparing) mainly used ecological arguments, claiming that protected areas are most beneficial for biodiversity conservation. The view that protected areas are most beneficial for biodiversity has also been pointed out by scientists (Filyushkina et al. 2022). The stakeholders sharing this view included regional administrations (represented by their experts in conservation planning), several municipalities, and the environmental organisation. The arguments from these stakeholders show that they consider biodiversity conservation as important but it is never clearly stated whether this is because they believe that biodiversity has an intrinsic and/or an instrumental value. Similar views were expressed in Sandström et al. (2016), where environmental organisations argued for protection of forest and motivate biodiversity conservation with the intrinsic value of biodiversity. One reason for the support for area protection by a wide range of stakeholders is probably the large area of forest in Sweden, which makes it possible to allocate different goals to different areas. In countries with a lower level of forest cover, this view would probably gain less support.

Previous studies have shown that environmental organisations often show low trust in conservation measures performed in managed stands and in the forest owners’ will to preserve biodiversity (Bjärstig et al. 2019). They often argue that land-sharing approaches including voluntary measures by forest owners and measures performed within certification schemes, such as the Forest Stewardship Council (FSC), are only a complement to land-sparing approaches such as formal protection. Small habitat patches like woodland key habitats, tree retention groups and small voluntary set asides are often viewed as only temporarily preserved and with greater risk of being logged (Bjärstig et al. 2019). Generally, they view measures taken both in Sweden and globally as insufficient to protect biodiversity (Bennett 2000; Ghazoul 2001; Elbakidze et al. 2011). This view probably contributes to the preference for area protection among environmental organisations. Also regional administrations, municipalities, and the forest company favoured a strategy with protected areas that are allocated where they are of most benefit for biodiversity or recreation. This is not completely in line with the Swedish forest policy, which acknowledges a high degree of voluntary measures, including both retention and area protection.

The forest owners’ association argued for conservation measures in managed stands focusing on forest owners’ property rights and freedom. Their representative also expressed a general reluctance to biodiversity conservation. This response express the values promoted by Swedish forest owners’ associations; previous studies show how forest owners’ associations have adopted a view that property rights are threatened, and both area protection and environmental consideration in managed forest are considered obstacles for economic activities and thus limiting forest owners’ freedom to choose how to manage their own forest (Hallberg-Sramek et al. 2020; Schneider et al. 2020; Sténs and Mårald 2020). For that reason, private forest owners are sometimes reluctant to both area protection and to apply biodiversity conservation measures voluntarily (Sandström et al. 2016; Eggers et al. 2020; Danley et al. 2021). This is especially the case for those who are members in Swedish forest owners’ associations; membership is correlated with a lower probability to leave unmanaged areas in forests (Danley et al. 2021). On the other hand, the forest owners’ association emphasized that ecosystem services, including those provided by biodiversity, need to be present where people live and on land that they use. This argument indicates a willingness to provide conservation measures in managed forest. However, non-industrial forest owners are a broad group, and in Sweden only 50% of them are members of forest owners’ associations. Among forest owners in Europe, the willingness to manage for biodiversity conservation varies, with forest owners being females, younger, with higher education, owning small properties and properties with high natural values being more willing to do so (Husa and Kosenius 2021; Tiebel et al. 2022). Thus, the argumentation provided by the owners’ association might not reflect the forest owners’ community as a whole. More detailed studies, e.g. including the demography and norms of forest owners’ associations, are needed to understand forest owners’ preferences for conservation strategies.

Expanding protected area networks might give raise to conflicts. From a perspective of justice, in the sense that forest owners should contribute equally to biodiversity conservation, it can be a challenge that conservation values are not evenly distributed, since that can result in protected areas to cover large parts of certain properties. Recently, the Swedish Government has affirmed the importance of private property right and suggested changes that results in more voluntary measures, e.g., by deciding that voluntary area protection should be the main measure to protect areas for the authorities (Anonymous 2021). However, a disadvantage with a system fully based on voluntary measures is that it is expensive, and makes it harder to protect larger areas and to protect the areas most important for biodiversity (Nieminen et al. 2021). Thus, the current policy is not in line with the preferences of a wide range of stakeholders, who in this study preferred more formal protection of sites selected based on their value for biodiversity.

The main argument for prolonged rotation (land-sharing) was that it results in a landscape better suited for recreation. This argument has also been used by scientists in a previous study (Filyushkina et al. (2022) and is consistent with research on recreationists’ stated preferences (Edwards et al. 2012). In contrast, representatives from regional administrations and municipalities highlighted that protected areas are important for recreation as being continuous areas of wilderness or easily accessable sites around areas with dense populations. In addition, prolonged rotations were motivated by one municipality suggesting that older forests contribute to the achievement of all goals (biodiversity, climate, and recreation). Thus, the arguments for prolonged rotations are mainly about the provision of ecosystem services (and thus anthropocentric), but also with a biodiversity conservation component.

An argument raised by the recreational organisation against area protection was that it could obstruct permission for activities such as sport events. Possible conflicts between recreation and area protection might also be due to that large protected areas might miss important infrastructure such as paths, that some areas might be closed for public, that there could be increased risks associated with old growth structures such as dead trees, or that high numbers of visitor could cause damage to natural values. In protected areas, usually infrastructure such as path networks and shelters are well developed and the natural values are appreciated by the visitors (Juutinen et al. 2011). This is also the case in other regions in Europe (Telbisz et al. 2023) and Asia (Hong-Kong) (Cheung and Fok 2014). In Scandinavia, strict reserves that do not allow public access are rare and mostly consist of temporal restrictions at the bird breeding season. The need to reduce access to protected areas (Coleman et al. 2013; Ngoprasert et al. 2017), and consequently also the risk of conflicts regarding activities in the area, are higher in more densely populated regions (Jones et al. 2016).

### Spatial allocation of conservation measures and protected areas

The motivation for concentrated allocation of protected areas expressed by regional administrations and the environmental organisation focused on the preservation of species, which is consistent with mainly ecocentric but also anthropocentric perspectives.. However, purely anthropocentric arguments also occurred, including practical considerations; the forest company claimed that it was more convenient to concentrate conservation measures to certain areas.

Practical considerations were also raised for spreading out conservation measures. One municipality and the forest owners’ association argued that it is easy and equitable to apply similar measures in all harvested stands. Furthermore, the forest owners’ association argued that a more even distribution of conservation measures implies that ecosystem services are more evenly delivered throughout the landscape. This argumentation exemplified an anthropocentric mind-set especially for the forest owners’ association.

Regarding allocation of conservation measures, there might be both conflict and synergies between stakeholders that represent recreation and biodiversity conservation. Setting aside forest for recreation close to areas with a dense population might not overlap with the areas most valuable for biodiversity, and recreational activities might be negative for biodiversity as an effect of overcrowding and littering (Niemelä et al. 2005; Nousiainen and Mola-Yudego 2022). Different recreationists prefer different forest attributes (Juutinen et al. 2011). The preferences of those wanting large wilderness areas are similar to preferences for stakeholders preferring large protected areas for biodiversity conservation, which implies there are possible synergy effects.

### Size of protected areas

As expected and similar to the argumentation for the protection of forest discussed above, several regional administrations and the environmental organisation preferred a few large protected areas rather than many small ones. The arguments for large protected areas focused on species conservation and consistent with arguments from experts; long-term species persistence becomes higher with a few larger areas set aside (Filyushkina et al. 2022). More surprising is the preference for large reserves by the forest company supported by the argument that this simultaneously benefit conservation and forest management. This is consistent with a scenario analysis by Eggers et al. (2020), which showed that a forest company included larger parts of the land as protected areas compared with a forest owner’s association, possibly supporting the view that it is more efficient to spatially separate conservation and management for large forest owners. The motivations for smaller protected areas were mainly anthropocentric. A few municipalities argued that it is better with many small set asides situated close to towns to allow recreation activities.

## Conclusions for management and governance implications

In our study, the anthropocentric mind-set shown by the arguments put forward by a forest owners’ association and a recreational organisation, and the arguments focusing on species conservation (which can be associated both with an anthropocentric and ecocentric mindset) put forward by regional administrations, and the environmental organisation, clearly reflect how the stakeholders’ underlying values influence which conservation strategy they prefer. The forest owner’s association and the recreational organisation are formed around the use of certain ecosystem services provided by forests, which can explain their more anthropocentric mindset, in comparison to other stakeholders, which are not to the same extent directly dependent on such ecosystem services. We found that more focus on the use of ecosystem services tends to generate a preference for land sharing, since that limits the use of forests to less extent. In contrast, land sparing are preferred by those focusing more on species conservation. These different views might polarize the debate and constitute obstacles in an adaptive collaborative process (Johansson et al. 2018; Bjärstig et al. 2019). Our results show that stakeholders use a wide palette of arguments when making decisions about conservation strategies. This implies that different values can lead to similar preferences, at least if conservation efforts does not come at the expense of human interests. Understanding values and arguments from different stakeholders can help to gain support for biodiversity conservation by tailoring information to groups with different values, e.g., by using economic and ecosystem service based argument when communicating with anthropocentric stakeholders.

The large number of arguments might indicate that many stakeholders are willing to learn and discuss pros and cons of different alternatives. There is thus a potential to create platforms where stakeholders can meet and learn from each other, e.g., by applying a structured and adaptive collaborative process (Johansson et al. 2018). Such a process can mitigate problems with closed groups that repeat the organisations’ view while being reluctant to new and opposite views and arguments (so-called echo-chambers according to Sténs and Mårald (2020)). In a Swedish context, where collaboration including different stakeholders is a key feature of current forest governance, it is relevant to address the stakeholders’ underlying goals and arguments at an early stage. This can reveal competing views on the values and preferences surrounding nature conservation and open up for possibilities to address synergies and trade-offs despite their different approaches (see, e.g., Emerson and Nabatchi 2015; Hurlbert and Gupta 2015).

The ‘most preferred’ and ‘least preferred’ scenarios varied among stakeholders and sometimes one’s best scenario was the worst scenario for another stakeholder. These conflicting preferences, together with the limited trust among environmental organisations, forest owners, and authorities (Bjärstig et al. 2019) can be an obstacle for efficient landscape planning. In the current Swedish context, with a large proportion of the forest managed for sustained yield and a forest policy that promotes wood production and “more of everything” (Beland Lindahl et al. 2017), an approach combining several conservation measures is probably the most efficient. A combined approach could add the benefits of large protected areas to support biodiversity and recreation with conservation in managed stands that support local biodiversity and pin-point small biodiversity hotspots. Such a compromise can probably also gain support by a wide range of stakeholders. Further, an analysis of the trade-offs between financial outcomes and ecological indicators has suggested that a mixture of several managements regimes is better at balancing conflicting objectives (Eggers et al. 2020). A combined approach would also allow for different measures to be implemented depending on the owner category. Large protected areas could preferably be based on state or company owned land while private forest owners could contribute with measures for biodiversity conservation in managed stands. To achieve this, a forest policy where voluntary measures are promoted, including relevant subsidies for prioritized measures, is crucial.

### Supplementary Information

Below is the link to the electronic supplementary material.

Supplementary file1 (PDF 3917 KB)
