# Supplementary material for: “Land-sparing benefits biodiversity while land-sharing benefits ecosystem services”: Stakeholders’ perspectives on biodiversity conservation strategies in boreal forests
Source: Ambio. 2023 Oct 11;53(1):20–33. doi: 10.1007/s13280-023-01926-0 (PMC10692042; doi:10.1007/s13280-023-01926-0)
Supplement: Supplementary file 1 — Supplementary file1 (PDF 3917 KB) [file 13280_2023_1926_MOESM1_ESM.pdf]

Ambio

Electronic supplementary material

This supplementary information has not been peer reviewed.

Title: **“Land-sparing benefits biodiversity while land-sharing benefits ecosystem services” - stakeholders’ perspectives on biodiversity conservation strategies in boreal forest**

Authors: Therese Löfroth*, Sonia Merinero*, Johanna Johansson, Eva-Maria Nordström, Emma Sahlström, Jörgen Sjögren, Thomas Ranius

*authors contributed equally

Supplementary material S1

Figure S1. The seven scenarios simulating the allocation of forest stands and their output for stand age, large trees and deadwood to conservation and timber production in land-sparing (1A, 1B, 2A, 2B) and land-sharing strategies (3A, 3B, 4) in a typical boreal forest landscape. The scenarios were presented to the respondents together with the questionnaire (S2).

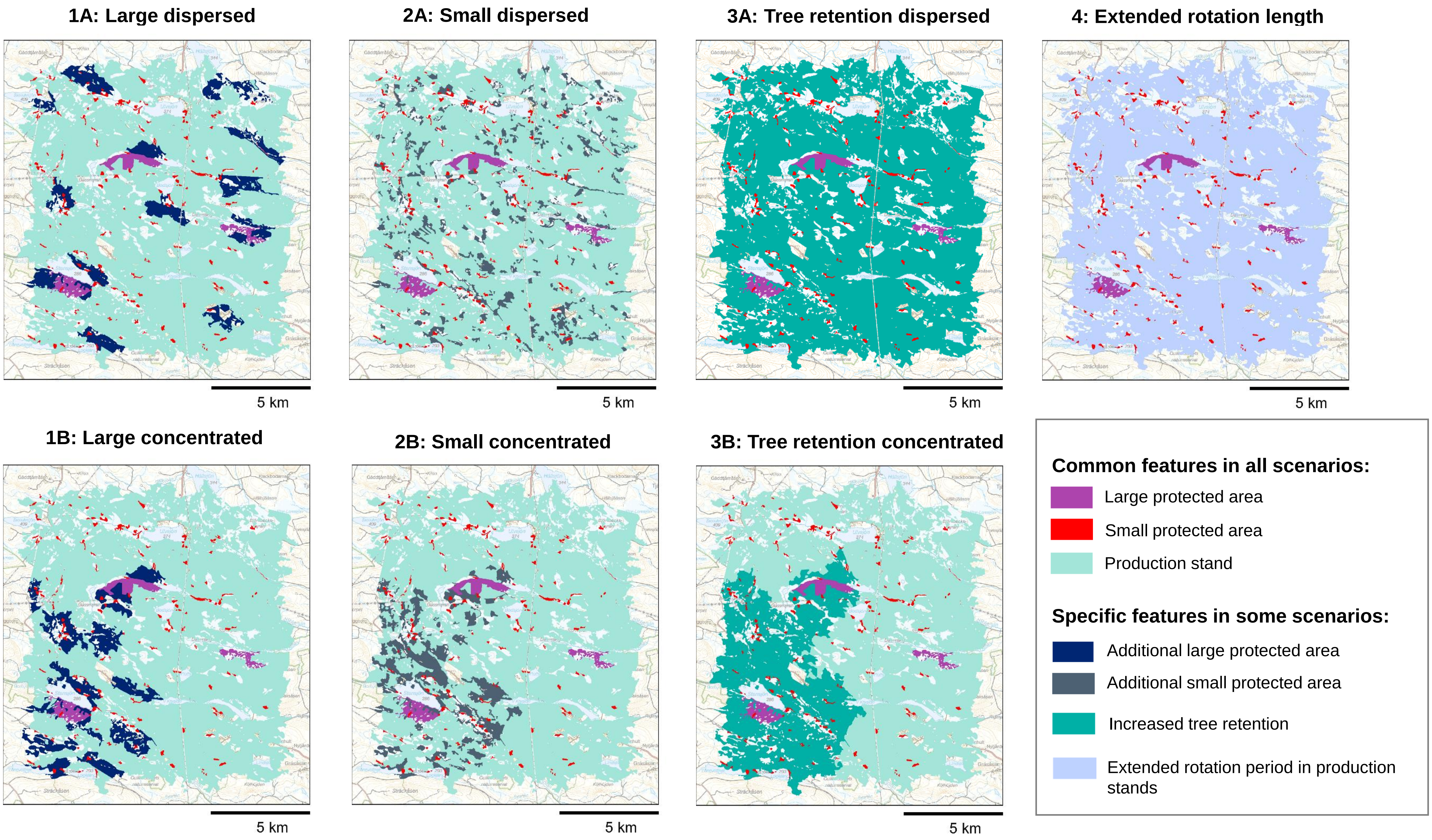
1A: Large dispersed
2A: Small dispersed
3A: Tree retention dispersed
4: Extended rotation length
1B: Large concentrated
2B: Small concentrated
3B: Tree retention concentrated
5 km
Common features in all scenarios:
Large protected area
Small protected area
Production stand
Specific features in some scenarios:
Additional large protected area
Additional small protected area
Increased tree retention
Extended rotation period in production stands

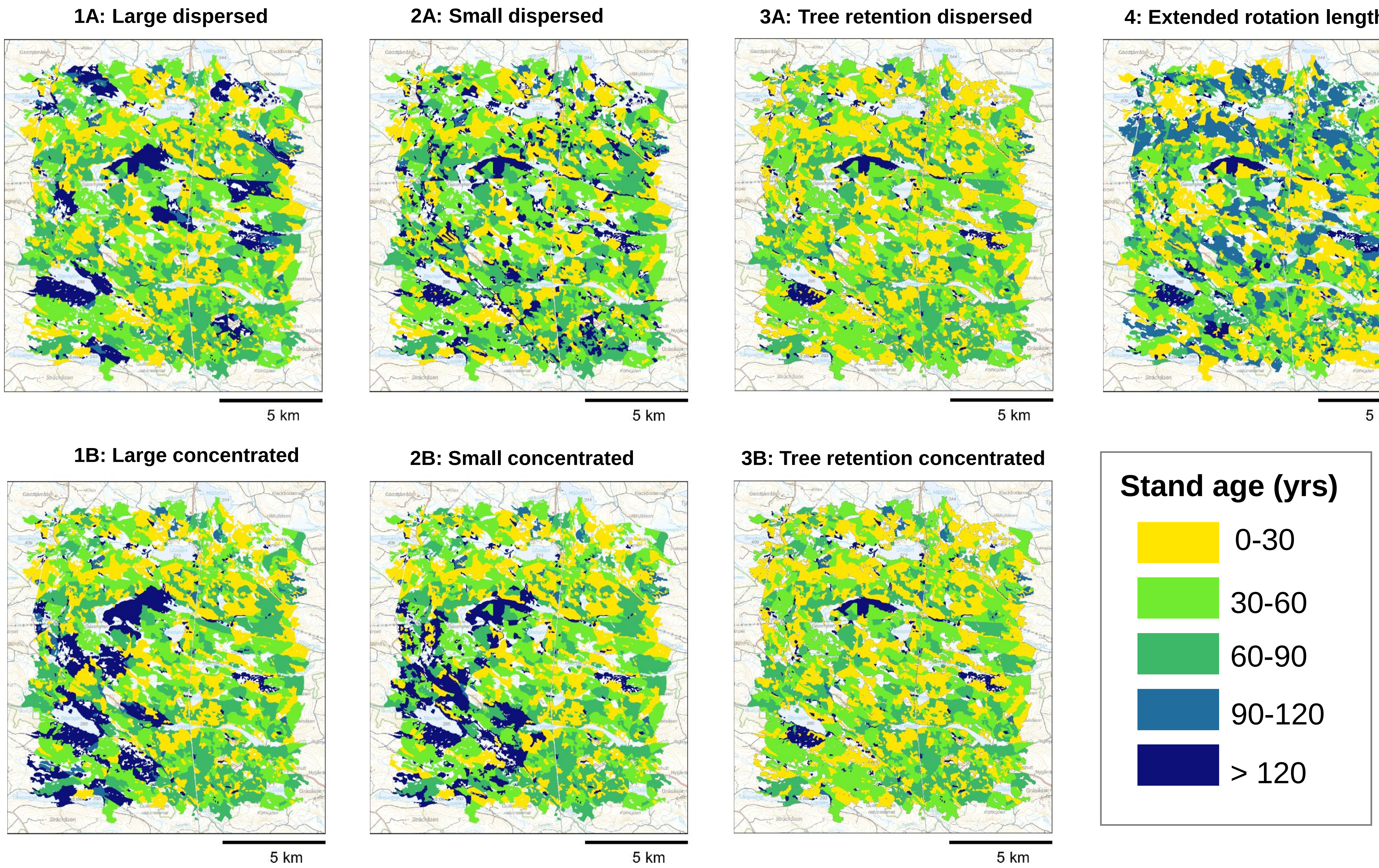
1A: Large dispersed
2A: Small dispersed
3A: Tree retention dispersed
4: Extended rotation length
1B: Large concentrated
2B: Small concentrated
3B: Tree retention concentrated
5 km
Stand age (yrs)
0-30
30-60
60-90
90-120
> 120

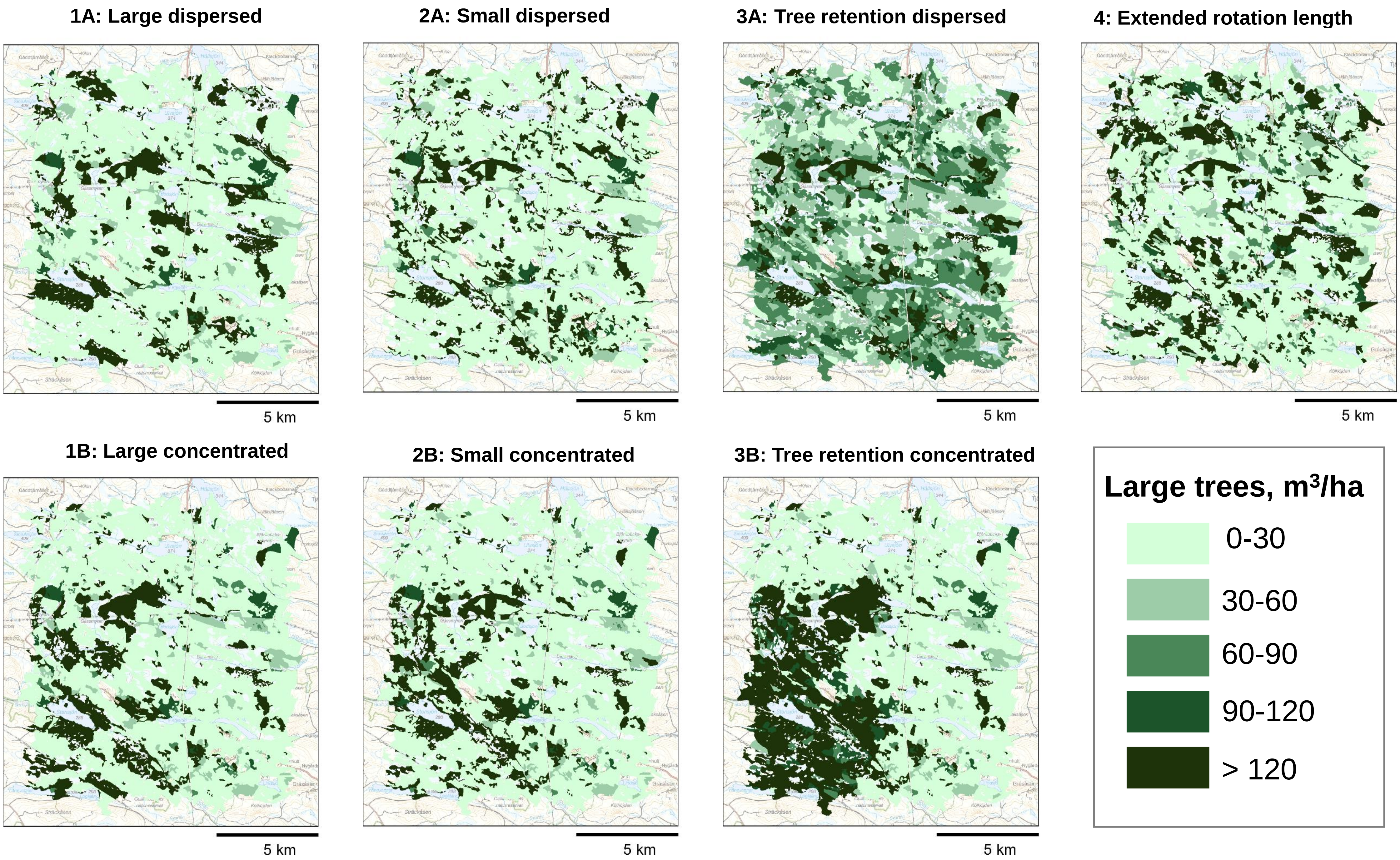
1A: Large dispersed
2A: Small dispersed
3A: Tree retention dispersed
4: Extended rotation length
5 km
5 km
5 km
5 km
1B: Large concentrated
2B: Small concentrated
3B: Tree retention concentrated
5 km
5 km
5 km
Large trees, m³/ha
0-30
30-60
60-90
90-120
> 120

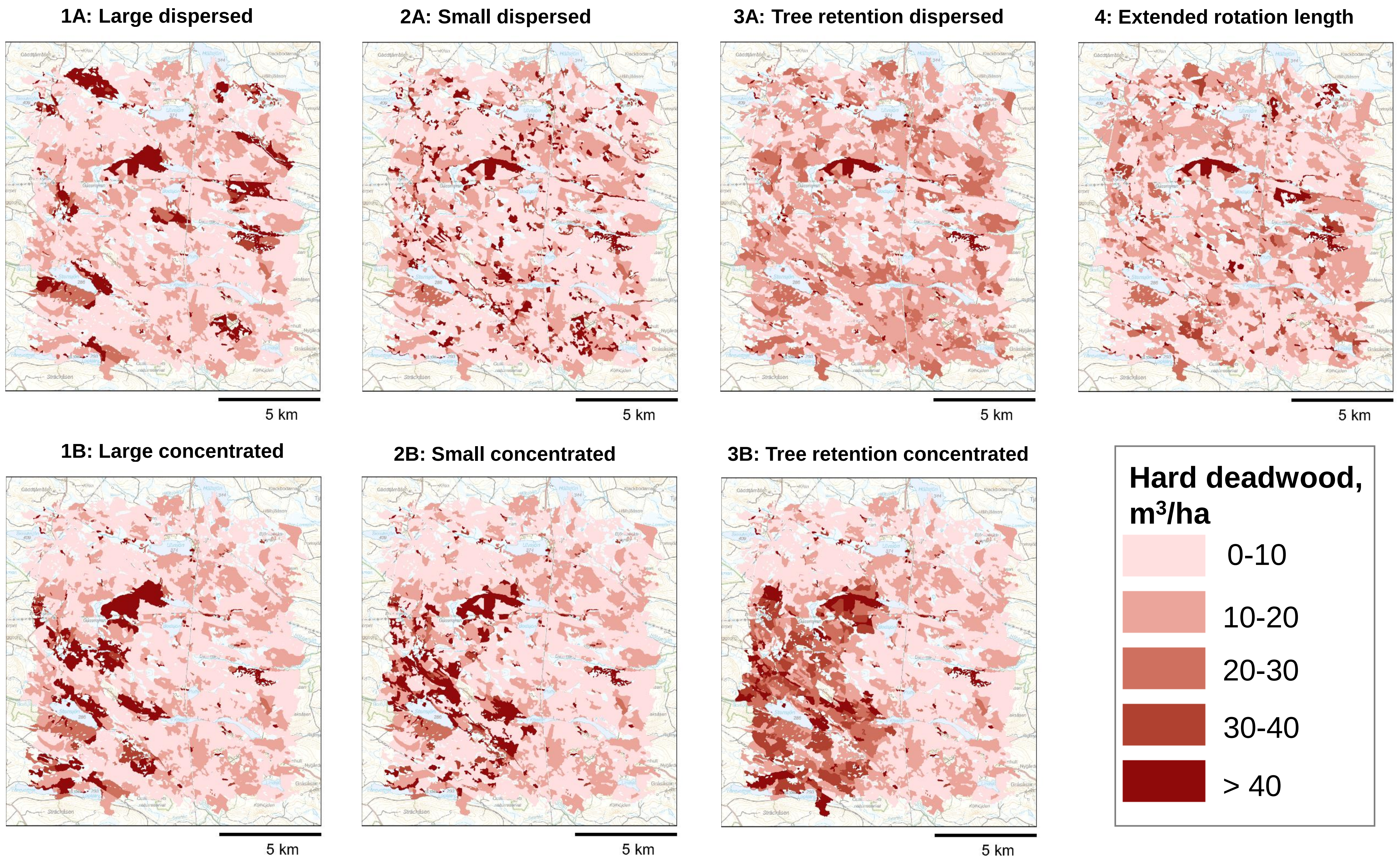
1A: Large dispersed
2A: Small dispersed
3A: Tree retention dispersed
4: Extended rotation length
1B: Large concentrated
2B: Small concentrated
3B: Tree retention concentrated
5 km
Hard deadwood, m³/ha
0-10
10-20
20-30
30-40
> 40

Supplementary material S2

SURVEY – Stakeholders' perspectives on landscape-scale biodiversity conservation strategies in boreal forests.

This survey addresses questions about the suitability of biodiversity conservation measures in forests according to the main goals of your organization. Before answering the questions, please read carefully the scenario descriptions available in the following link. The expected time to read the study description and filling this survey is 30 minutes.

We kindly ask you to evaluate the suitability of seven alternative scenarios that combine different conservation measures commonly used in Swedish forests. We have simulated these scenarios in a typical forest landscape in central Sweden to illustrate the differences in forest development among scenarios. Each scenario leads to different spatial configurations of three features relevant for biodiversity conservation: forest stand age, volume of large trees and volume of deadwood

We assume that the forestry system works as it does today in terms of ownership, legislation and economic conditions. These elements are out of the scope of this study. Moreover, our questions refer to the strategy that we should choose today and not about responses to changes in future forestry.

SECTION 1: Information about the organization and its representative:

Question 1: Name of organization: ___________________________

Question 2: Your role within the organization: __________________________

Question 3: Which are the main goals of your organisation? We have classified the most common goals into four categories. Please evaluate the priority of these goals within your organisation. You are also welcome to add goals not represented in these categories:

- Biodiversity conservation (not a goal, low priority goal, high priority goal)
- Production of wood and other commodities (not a goal, low priority goal, high priority goal)
- Promotion of recreation (including hunting, berry picking, tourism) (not a goal, low priority goal, high priority goal)
- Climate change mitigation (not a goal, low priority goal, high priority goal)
- Other (not a goal, low priority goal, high priority goal)

SECTION 2: Scenario preferences according to the organization goals.

Question 1: Considering the main goals of your organization, please evaluate the suitability of each scenario in a scale from “Very unsuitable” to “Very suitable”.

Question 2: Which is the “MOST DESIRABLE” scenario that best suits the current goals of your organization?

- 1 A (Large reserves – concentrated)
- 1 B (Large reserves – dispersed)
- 2 A (Small reserves – concentrated)
- 2 B (Small reserves – dispersed)
- 3 A (Retention trees – concentrated)
- 3 B (Retention trees – dispersed)
- 4 (Extended rotation period)

Question 3. Which are the main factors that motivate your answer? Please, mark as many reasons as you consider and add other reasons if needed.

☐ It benefits biodiversity conservation

☐ It enhances production of wood and other commodities

☐ It facilitates recreation activities

☐ It support climate change mitigation

☐ I do not have an answer

☐ Other

Question 4: Please, explain the reasoning behind these factors that made you choose the most desirable scenario?

Question 5: Which is the “LEAST DESIRABLE” scenario that worst suits the current goals of your organization?

- 1 A (Large reserves – concentrated)
- 1 B (Large reserves – dispersed)
- 2 A (Small reserves – concentrated)
- 2 B (Small reserves – dispersed)
- 3 A (Retention trees – concentrated)
- 3 B (Retention trees – dispersed)
- 4 (Extended rotation period)

Question 6. Which are the main factors that motivate your answer? Please, mark as many reasons as you consider and add other reasons if needed.

☐ It benefits biodiversity conservation

☐ It enhances production of wood and other commodities

☐ It facilitates recreation activities

☐ It support climate change mitigation

☐ I do not have an answer

☐ Other

Question 7: Please, explain the reasoning behind these factors that made you choose the most desirable scenario?

Question 8: If your organization were to assess the suitability of this kind of scenarios in a real-life project, which sources of information would be most important to support this assessment?

- Knowledge of the organization's professionals.
- Previous working experience of the organization on similar projects.
- Official reports from public administration.
- Experts’ opinions (outside your organisation).
- Scientific literature
- Other: _____________

Question 9: Please, can you list the most relevant examples of these sources of information?
